# Spastic paraplegia leading PSEN1‐related familial Alzheimer’s disease in Italy

**DOI:** 10.1002/alz.089719

**Published:** 2025-01-03

**Authors:** Arun Meyyazhagan, Preethi Basavaraju, Marialuisa Miele, Toshitaka Kawarai, Emanuele Panza, Antonio Orlacchio

**Affiliations:** ^1^ University of Perugia, Perugia Italy; ^2^ Bharathiar University, Coimbatore India; ^3^ Ospedale S. Giovanni Battista, Foligno (PG) Italy; ^4^ Hyogo Prefectural Harima‐Himeji General Medical Center, Himeji Japan; ^5^ University of Bologna, Bologna Italy; ^6^ IRCCS Azienda Ospedaliero‐Universitaria di Bologna, Bologna Italy; ^7^ IRCCS Fondazione Santa Lucia, Rome Italy

## Abstract

**Background:**

We researched the occurrence, neuropathology, and clinical features of spastic paraplegia (SP) associated to dementia in presenilin 1 (PSEN1) Italian patients related to familial Alzheimer’s disease (AD).

**Methods:**

We carried out whole exome sequencing in 33 familial AD probands with hereditary spastic paraplegia (HSP) that resulted negative for the identification of pathogenetic variants in known HSP genes. One patient was identified with a DNA variant in PSEN1, and bioinformatic analysis was conducted to characterize its pathogenetic nature. Furthermore, brain biopsy was executed once he deceased. Bioinformatic analysis was conducted to characterize the new variant.

**Results:**

This research yielded the identification of one novel PSEN1 pathogenic variant. Notably, the same affected individual revealed SP at 22 years of age and 23 years later developed AD. Amyloid deposition with diffuse cortical plaques was described on brain biopsy. Differently from previous reports, we found that Italian PSEN1 patients displaying primarily as SP have an earlier age of onset and are associated with mutations located before codon 200.

**Conclusion:**

The outcomes of this study increase the clinical and mutational landscape associated with PSEN1‐related familial AD. Expanding results of different populations, we strongly recommend PSEN1 genetic testing in affected subjects having SP with no variants in known HSP‐related genes, mostly when linked with a family history of AD.

